# Comparative pharmacokinetics of four major compounds after oral administration of Mori Cortex total flavonoid extract in normal and diabetic rats

**DOI:** 10.3389/fphar.2023.1148332

**Published:** 2023-03-01

**Authors:** Shan Xiong, Xiaofan Li, Haiping Chu, Zhipeng Deng, Linying Sun, Jia Liu, Yanling Mu, Qingqiang Yao

**Affiliations:** ^1^ School of Pharmacy and Pharmaceutical Sciences & Institute of Materia Medica, Shandong First Medical University & Shandong Academy of Medical Sciences, NHC Key Laboratory of Biotechnology Drug (Shandong Academy of Medical Sciences), Key Lab for Rare and Uncommon Diseases of Shandong Province, Jinan, China; ^2^ Institute of Basic Medicine, Shandong First Medical University & Shandong Academy of Medical Science, Jinan, China; ^3^ School of Pharmaceutical Sciences, Shandong University of Traditional Chinese Medicine, Jinan, China; ^4^ School of Public Health, Shandong First Medical University & Shandong Academy of Medical Science, Jinan, China

**Keywords:** UPLC-MS/MS, pharmacokinetic, Mori Cortex total flavonoid extract, diabetes mellitus, rats

## Abstract

**Introduction:** Mori Cortex has been used in traditional Chinese Medicine as an antidiabetic agent. The aim of this study was to establish a UPLC-MS/MS method for simultaneous determination of morin, morusin, umbelliferone and mulberroside A in rat plasma and investigate the pharmacokinetics differences between normal and diabetic rats following oral administration of Mori Cortex total flavonoid extract.

**Methods: **Samples were pre-treated by protein precipitation and genkwanin was used as internal standard. Chromatographic separation was performed using a Hypersil GOLD C_18_ column (50 mm × 2.1 mm, 3 μm). The mobile phase consisted of acetonitrile and water (containing 0.1% formic acid) in gradient mode at a flow rate of 0.5 ml/min. The transitions of m/z 300.9→107.1, m/z 419.3→297.1, m/z 160.9→77.0, m/z 567.1→243.2 and m/z 283.1→268.2 were selected for morin, morusin, umbelliferone, mulberroside A and internal standard, respectively.

**Results:** The intra- and inter-day precision for analytes were less than 12.5% and the accuracy ranged from −8.1% to 3.5%. The extraction recovery was >88.5% and no obvious matrix effect was observed. The *AUC*_(0-t)_ and *C*_max_ of morin were 501.3 ± 115.5 ng/mL*h and 127.8 ± 56.0 ng/mL in normal rats and 717.3 ± 117.4 ng/ml*h and 218.6 ± 33.5 ng/ml in diabetic rats. Meanwhile, the *AUC*_(0-t)_ and *C*_max_ of morusin were 116.4 ± 38.2 ng/ml*h and 16.8 ± 10.1 ng/mL in normal rats and 325.0 ± 87.6 ng/mL*h and 39.2 ± 5.9 ng/ml in diabetic rats. For umbelliferone and mulberroside A, the *AUC*_(0-t)_ and *C*_max_ also increased significantly in diabetic rats (*p* < 0.05).

**Discussion:** The validated method was successfully applied to the pharmacokinetic study in normal and diabetic rats.

## 1 Introduction

A large number of studies have shown that long-standing serious hyperglycemia is the main cause of metabolic disorders and autoimmune disorders (Maritim et al., 2003). Diabetes mellitus (DM) is a chronic disease caused by acquired deficiency in production of insulin by the pancreas, or by the ineffectiveness of the insulin produced (Riaz, 2009). This deficiency results in increased concentrations of glucose in the blood, which in turn leads to retinopathy, nephropathy, neuropathy, coronary heart disease, cerebrovascular disease, and peripheral vascular diseases (Gregg et al., 2016; O'Brien and Corrall, 1988). The main purpose of diabetes treatment is to prevent or delay the complications by improving blood sugar control (Kooti et al., 2016). In China, traditional Chinese medicine (TCM) has been widely used in the treatment of diabetes and its complications (Tong et al., 2012). The prevention and treatment of diabetic complications by using TCM have lots of advantage including comprehensive treatment and small toxicity and side effects (Jia et al., 2003; Wang et al., 2016; Martel et al., 2017; Aras et al., 2019).

Mori Cortex, also called “Sang-Bai-Pi” in Chinese, which is derived from the root bark of Morus *alba* L. according to the China Pharmacopeia (The Committee of China Pharmacopeia, 2020). The modern pharmacological studies have shown that Mori Cortex has the active effect of antidiabetic (Ma et al., 2018), antioxidant (Ahmad et al., 2013; Abbasa et al., 2014; Bayazid et al., 2020), anti-inflammatory (Lim et al., 2013; Bayazid et al., 2020), antimicrobial (Grienke et al., 2016) and anticarcinogenic (Lim et al., 2014). It was first recorded for the antidiabetic effect of Mori Cortex in “Compendium of Materia medica”. According to ancient prescriptions, the decoction of Cortex Mori (12 g) and Lycii Fructus (15 g) was used to control the blood glucose level for diabetic patients (Xiao et al., 2014). Recent studies have shown that Mori Cortex extract could lower the blood glucose and improve insulin resistance (Qi et al., 2016; Kim and Choe, 2017; Ma et al., 2018).

**FIGURE 1:**
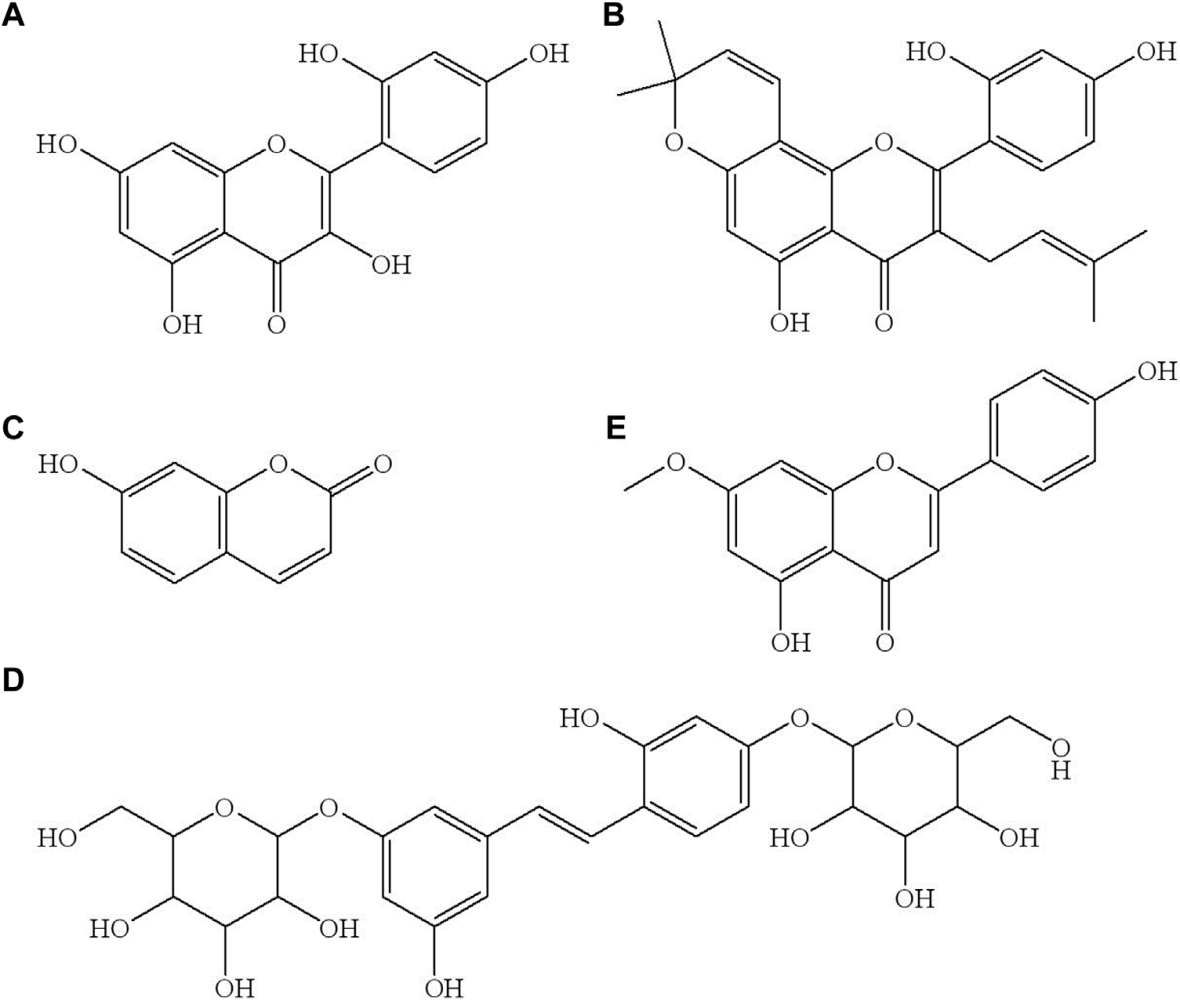
The chemical structure of morin **(A)**, morusin **(B)**, umbelliferone **(C)**, mulberroside A **(D)** and genkwain IS, **(E)**.

To the best of the authors’ knowledge, there is no study focusing on the simultaneous quantification of morin, morusin, umbelliferone, and mulberroside A in rat plasma. The aim of this study was to develop an ultra-performance liquid chromatography-tandem mass spectrometry (UPLC-MS/MS) method for the simultaneous determination of morin, morusin, umbelliferone and mulberroside A, which are the main active components of Mori Cortex total flavonoid extract with higher content, in normal and diabetic rat (Liu et al., 2019). The structures of these four target analytes and IS are shown in Figure 1.

## 2 Materials and methods

### 2.1 Chemicals and reagents

Mori Cortex total flavonoid extract (Lot number HP20171011), morin (98% purity), morusin (98% purity), umbelliferone (98% purity) and mulberroside A (98% purity) were supplied by Baoji Herbet Bio-Tech Co. Ltd (Baoji, China). Genkwanin (internal standard (IS), 98% purity) was supplied by the National Institute for the Control of Pharmaceutical and Biological Products (Beijing, China). Propylene glycol was purchased from Nanjing Weier Pharmaceutical Co. Ltd (Nanjin, China). Streptozotocin (STZ) was purchased from Sigma (Sigma-Aldrich, St Louis, MO, United States). HPLC-grade formic acid was obtained from Tianjin Kermel Chemical Reagent Co. Ltd (Tianjin, China). HPLC-grade acetonitrile and methanol were obtained from Tedia Company (Fairfield, United States). Purified water was employed by Wahaha Co. Ltd (Hangzhou, China). All of the other chemicals were analytical grade or better.

### 2.2 Animals

Male Sprague-Dawley rats (7–8 weeks old, weighing 200 ± 20 g) were purchased from Jinan Pengyue Experimental Animal Breeding Co. Ltd (Jinan, China). They were housed in a room with a 12/12 h light/dark cycle and an ambient temperature of 23°C ± 3°C. All animal experiments were carried out according to the National Institute of Health Guideline for the Care and Use of Laboratory Animals, and performed by the Animal Ethics Committee of School of Pharmacy and Pharmaceutical Sciences & Institute of Materia Medica, Shandong First Medical University & Shandong Academy of Medical Sciences (2017036, Jinan, China).

The rats were given a single intraperitoneal injection with a freshly prepared solution of STZ (60 mg/kg) in 0.1 mol/L citrate buffer (pH = 4.4) to induce diabetes (Seke Etet et al., 2017; Zhang et al., 2017). After 8 weeks, the rats with fasting blood glucose (FBG) for 5–6 h exceeding 16.7 mmol/L were considered successful diabetes models. The FBG was measured from the tail vein using a One-Touch Ultra® Blood Glucose Meter (LifeScan Inc., Milpitas, United States).

### 2.3 Instrumentation and UPLC-MS/MS conditions

A Shimadzu Prominence UPLC (Shimadzu, United States) system coupled to an AB SCIEX™ 5500 Q-Trap® mass spectrometer (Applied Bio-systems, United States) equipped with an electrospray ionization interface operated in negative multiple reaction monitoring (MRM) mode were applied to analysis. The analytes separation was achieved *via* gradient elution of 0.1% formic acid in water (A) and acetonitrile (B) at a flow rate of 0.5 mL/min on a Thermo Hypersil Gold C_18_ column (50 mm × 2.1 mm, 3 μm; Thermo Scientific, New York, United States). The run time was 4.0 min for each analysis. The gradient elution program was used as follows: 5% B→10% B at 0–0.2 min, 10% B→70% B at 0.2–2.0 min, 70% B at 2.0–2.5 min, 70% B→5% B at 2.50–2.51 min; 5% B at 2.51–4.0 min. The temperatures of autosampler and column were set at 15°C and 40°C, respectively. The supernatant injection volume was 2 μl. The MRM conditions (source-dependent mass parameters) were defined as follows: Ion Spray Voltage, −4500 V; temperature, 550°C; curtain gas, 35.0 psi; collision gas, medium; Gas 1, 55.0 psi; Gas 2, 55.0 psi. The transitions of m/z 300.9→107.1, m/z 419.3→297.1, m/z 160.9→77.0, m/z 567.1→243.2 and m/z 283.1→268.2 were selected for morin, morusin, umbelliferone, mulberroside A and IS, respectively. The system control and data analysis were performed using AB SCIEX Analyst software (version 1.6.3).

### 2.4 Preparation of standard solutions, calibration standards and quality control samples

The analytes were accurately weighted and separately dissolved in methanol to yield the stock solutions with a concentration of 5 mg/mL. The stock solutions were stored at −80°C until analyzed. The stock solutions were stepwise diluted with acetonitrile to make a series of mixed working solutions at concentration levels of 20–20000 ng/ml for target analytes. In addition, the IS was dissolved in methanol and then diluted with acetonitrile to obtain a working solution of 50 ng/ml. The solution of IS was maintained at 4°C.

Calibration standards were prepared by adding 10 μl of the mixed working solutions to 190 μl blank plasma to obtain final concentrations in the range 1–1000 ng/mL for target analytes. The quality control (QC) samples were prepared separately by the same method at four concentration levels of 1 (lower limit of quantification, LLOQ), 3 (LQC), 80 (MQC) and 800 ng/ml (HQC) for the four analytes.

### 2.5 Sample preparation

50 μl of rat plasma was mixed with 50 μl of IS solution (50 ng/ml) in a 1.5 ml polypropylene tube and then 100 μl acetonitrile was added to precipitate protein. The mixture was vortexed for 5 min, then centrifuged at 13000 rpm for 5 min at 4°C. The supernatant was collected in an autosampler vial and 2 μl of aliquot was injected into the UPLC-MS/MS system for analysis.

### 2.6 Validation of UPLC-MS/MS analytical method

The selectivity, linearity range, carryover, inter- and intra-day precision and accuracy, matrix effect, recovery and stability under different storage conditions were evaluated according to the FDA Guidance for Industry Bioanalytical Method Validation (Xiong et al., 2017; Food and Drug Administration, 2018; Li et al., 2019).

The selectivity was evaluated by comparing the chromatogram of blank rat plasma from six different matrices with standard plasma samples spiked with analytes and IS. The purpose was to explore whether there was interference from endogenous substances.

The linearity for morin, morusin, umbelliferone and mulberroside A were evaluated by plotting the peak area ratios of the analytes/IS versus the concentration values of the standard plasma samples on three consecutive days (a weighted 1/*X* ^2^ least squares linear regression). The LLOQ was determined by spiking the lowest concentration on the calibration curve with acceptable precision of less than 20% of the relative standard deviation (RSD, %) and relative error (RE, %) of ±20%. The carryover was assessed by analyzing the response of the blank plasma following the upper limits of quantification (ULOQ).

The intra- and inter-day accuracy and precision were determined by analyzing five replicates on three consecutive days. The intra-day assessment was investigated in the 1 day and the inter-day assessment was investigated for three consecutive days. The precision was calculated in terms of RSD (%), while the accuracy was expressed as the RE (%). The RSD should be within 15% and accuracy was required to not be exceed ±15% at four QC levels.

The recovery of four target analytes were evaluated at three QC levels (LQC, MQC, and HQC) by comparing the mean peak areas of QC samples in six replicates with that of the pre-extraction blank plasma spiked with the corresponding working standard solution. The matrix effect was investigated by comparing the peak area of analytes resolved in pre-extraction matrix of blank plasma with those in the water-substituted samples. The recovery and matrix effect of the IS were assessed in the same way.

The stability of analytes in rat plasma was assessed by analyzing QC samples at low, middle and high levels (*n* = 5) under different storage condition including the short-term stability at room temperature for 6 h, the post-treatment stability at 15°C in autosampler for 12 h, three freeze-thaw stability, and the long-term stability at −80°C for 7 days.

### 2.7 Preparation of the oral solutions

The Mori Cortex total flavonoid extract (8 g) was stirred with propylene glycol (20 ml) by ultrasonic in a 50 ml volumetric flask, and then added purified water to 50 ml slowly. The contents of morin, morusin, umbelliferone and mulberroside A in the Mori Cortex total flavonoid extract were 0.1093%, 1.0050%, 0.1128% and 0.0764%, respectively.

### 2.8 Pharmacokinetic study

Ten male Sprague-Dawley rats were enrolled in the pharmacokinetic study: five normal rats and five diabetic rats. All of the rats were fasted for at least 12 h and had free access to drinking water before the experimental. The two groups of rats were administered Mori Cortex total flavonoid extract at the doses of 2 g/kg by oral after overnight fasting. Blood samples (150 μl) were obtained from retroorbital plexus into a heparinized tube at 0 (pre-dose), 0.083, 0.167, 0.5, 0.75, 1, 1.5, 2, 3, 5, 8, 12 and 24 h after oral administration. The collected blood samples were immediately centrifuged at 3500 rpm at 4°C for 15 min to obtain plasma fraction. The samples were frozen at −80°C until analyzed.

## 3 Results

### 3.1 Method development

Acetonitrile/methanol-water (containing 0, 0.1% formic acid and 10 mM ammonium acetate with 0.1% formic acid) being the mobile phase with gradient elution was used to evaluate UPLC separation and sensitivity in MS detection in order to obtain the good peak shape and optimal response for all the analytes. Finally, the mobile phase containing acetonitrile and 0.1% formic acid in water was selected for the separation of the four analytes and IS, and the excellent peak shape and lower background noise were also obtained.

The ESI source was operated in negative ion mode to achieve maximum response and the deprotonated precursor molecular ions [M−H]^−^ were chosen to be monitored. The m/z 300.9→107.1 as the quantitative ion for morin was selected due to the better peak shape of LLOQ samples. For morusin, the mass transition of m/z 419.3→89.0 was not chosen for quantitative analysis because it showed interference of endogenous substances in blank plasma samples. The MS/MS spectrogram of morin, morusin, umbelliferone, mulberroside A and IS are shown in Supplementary Figure S1. The optimal mass spectrometry parameters and transitions for analytes are listed in Table 1.

**TABLE 1:** Optimized mass parameters for analytes and IS.

Analytes	Precursor ion (m/z)	Quantitative ion (m/z)	Qualitative ion (m/z)	Dp (V)	Ep (V)	Cxp (V)	CE (V)
Morin	300.9	107.1	125.0	−20	−15	−15	−35
Morusin	419.3	297.1	89.0	−60	−15	−43	−20
umbelliferone	160.9	77.0	105.0	−100	−15	−5	−34
mulberroside A	567.1	243.2	405.2	−200	−10	−5	−39
IS	283.1	268.2	239.9	−65	−15	−50	−32

### 3.2 Method validation

**FIGURE 2:**
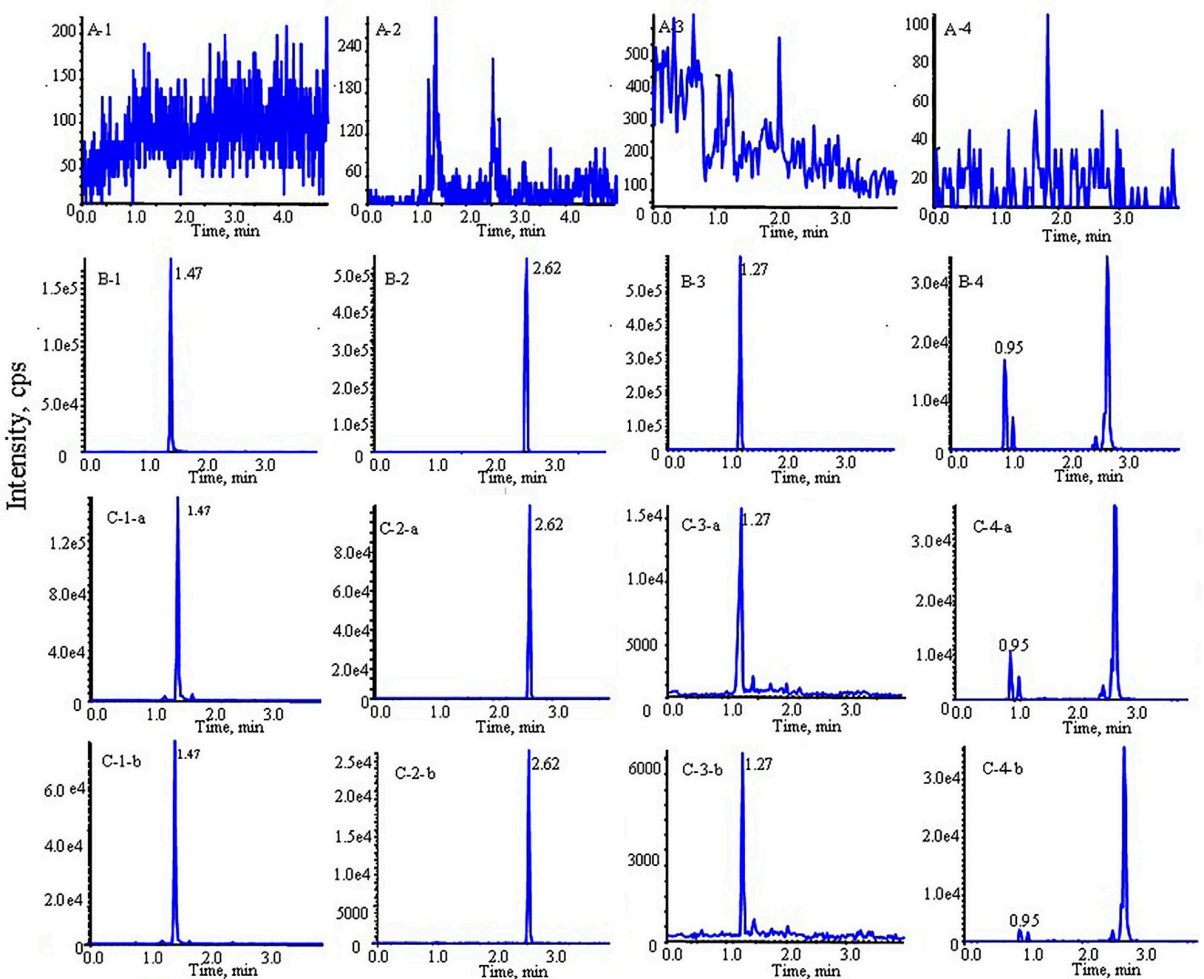
Typical multiple reaction monitoring (MRM) chromatograms of morin, morusin, umbelliferone and mulberroside A from rat plasma: **(A)** blank plasma sample; **(B)** blank plasma spiked with analytes; **(C)** rat plasma sample after oral administration of Mori Cortex total flavonoid extract (1: morin, 2: morusin, 3: umbelliferone, 4: mulberroside A; **(a)** diabetic rats, **(b)** normal rats).

As shown in Figure 2, the retention times were 1.47, 2.62, 1.27, 0.95 and 2.05 min for morin, morusin, umbelliferone, mulberroside A and IS, respectively. No unacceptable interference was observed at the retention times.

The eight-point calibration curve was found to be linear over the concentration range of 1–1000 ng/mL for four analytes. Correlation coefficients were ≥0.99 for all analytes. The calibration curve results for four analytes are summarized in Table 2. The LLOQ values for analytes were 1 ng/mL with RSD <12.5% and RE varied from −5.6% to 3.5% (Table 3), indicating LLOQs of all analytes met the requirements. There was no carryover effect after the injection of ULOQ samples.

**TABLE 2:** analytical parameters for quantification of analytes.

Analytes	Regression equation	*R*	Linear range (ng/mL)	LLOQ (ng/mL)
Morin	*y* = 2.98 × 10^−4^ *x*+9.24 × 10^−4^	0.9967	1–1000	1
Morusin	*y* = 1.22 × 10^−3^ *x*+1.19 × 10^−3^	0.9964	1–1000	1
umbelliferone	*y* = 2.03 × 10^−3^ *x*+1.51 × 10^−3^	0.9978	1–1000	1
mulberroside A	*y* = 8.18e^−5^ *x*+1.64e^−5^	0.9961	1–1000	1

**TABLE 3:** Precision and accuracy for the determination of analytes in rat plasma (*n* = 5).

Analytes	Spiked concentration (ng/mL)	Intra-day		Inter-day	
		Precision (RSD,%)	Accuracy (RE,%)	Precision (RSD,%)	Accuracy (RE,%)
Morin	1	5.7	−4.2	9.4	−1.9
	3	5.5	−6.3	5.4	−7.5
	40	5.7	−2.5	7.9	−6.6
	800	4.1	−0.5	5.9	−2.9
Morusin	1	9.5	3.5	12.5	−5.4
	3	2.4	−3.3	6.4	−8.1
	40	5.5	−3.9	7.6	−1.5
	800	5.0	−3.0	7.8	2.7
umbelliferone	1	5.8	−0.3	7.8	−5.6
	3	4.6	1.8	6.4	−1.4
	40	5.1	−0.7	5.8	1.6
	800	5.2	−4.1	7.6	−2.1
mulberroside A	1	8.9	−0.9	11.0	−3.2
	3	6.7	−0.5	9.0	−1.7
	40	8.5	3.1	10.4	−2.9
	800	4.7	−5.2	8.4	−2.0

The intra- and inter-day precision and accuracy within the acceptance limit for the analytes are summarized in Table 3. The precision was ≤10.4% and the accuracy was within ± 8.1% for four analytes. The data suggested that the analytical method was reliable and accurate.

The mean extraction recovery and matrix effect of the analytes and IS are summarized in Supplementary Table S1. The results suggested that the assay obtained high recovery and no obvious matrix effect of each analyte and IS was observed.

The stability data of four analytes are shown in Supplementary Table S2. The results proved that the storage conditions, disposal, intermittent analysis and analysis techniques were valid and reliable for the analytes in rat plasma.

### 3.3 Application to a pharmacokinetic comparison

**FIGURE 3:**
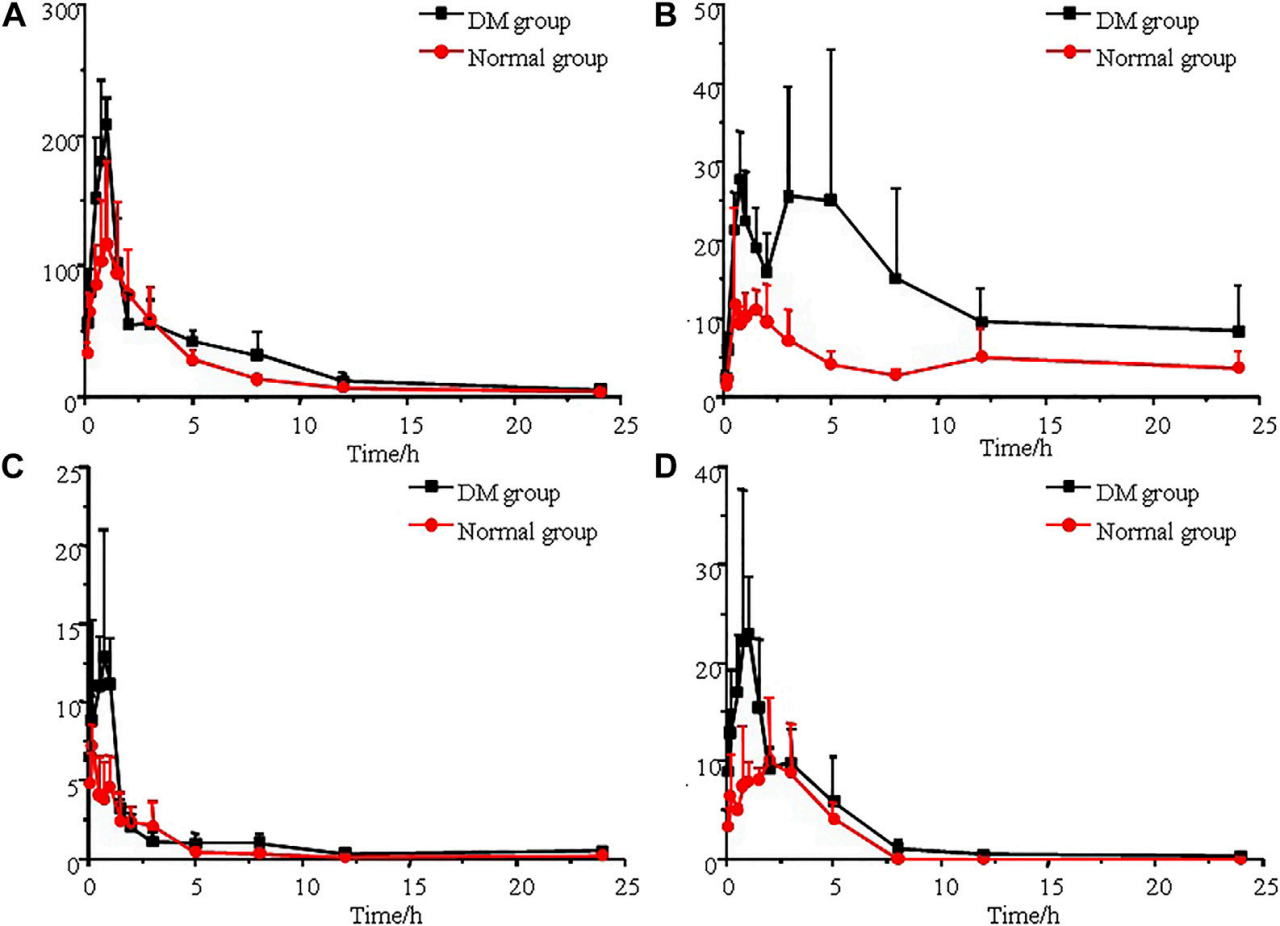
Mean plasma concentration-time curves of morin **(A)**, morusin **(B)**, umbelliferone **(C)** and mulberroside A **(D)** after oral administration of Mori Cortex total flavonoid extract (*n* = 5).

The established UPLC-MS/MS method was successfully applied to the determination of morin, morusin, umbelliferone and mulberroside A in rat plasma samples collected from normal rats and diabetic rats after oral administration of Mori Cortex total flavonoid extract. The mean plasma concentration versus time plots for morin, morusin, umbelliferone and mulberroside A are shown in Figure 3. In addition, the pharmacokinetic parameters of the normal and diabetic rats were calculated by DAS software version 2.0 and compared using the independent samples *t*-test (a value of *p* < 0.05 was considered statistically significant). Compared with normal rats, some main pharmacokinetic parameters of all target analytes obtained from diabetic rats changed. The *AUC*_(0-t)_ and *C*_max_ of morin were 501.3 ± 115.5 ng/ml*h and 127.8 ± 56.0 ng/mL in normal rats and 717.3 ± 117.4 ng/ml*h and 218.6 ± 33.5 ng/mL in diabetic rats. Additionally, the *AUC*_(0-t)_ and *C*_max_ of morusin were 116.4 ± 38.2 ng/ml*h and 16.8 ± 10.1 ng/mL in normal rats and 325.0 ± 87.6 ng/mL*h and 39.2 ± 5.9 ng/mL in diabetic rats. For umbelliferone and mulberroside A, the *AUC*_(0-t)_ were 16.4 ± 5.1 and 36.8 ± 14.6 ng/ml*h in normal rats, and 28.4 ± 3.6 and 75.6 ± 11.1 ng/ml*h in diabetic rats. Meanwhile, the *C*_max_ were 7.6 ± 0.7 and 13.1 ± 6.5 ng/mL in normal rats, and 16.8 ± 6.6 and 28.5 ± 12.3 ng/ml in diabetic rats. The changes were significant (*p* < 0.05). The main pharmacokinetic parameters are presented in Table 4.

**TABLE 4:** Pharmacokinetic parameters of analytes after oral administration of Mori Cortex total flavonoid extract in normal and diabetic rats plasma (mean ± SD, *n* = 5).

Pharmacokinetic parameters	Unit	Morin		Morusin		Umbelliferone		Mulberroside A	
		Normal rats	Diabetic rats	Normal rats	Diabetic rats	Normal rats	Diabetic rats	Normal rats	Diabetic rats
*AUC*_(0-t)_	ng/mL^a^h	501.3 ± 115.5	717.3 ± 117.4^a^	116.4 ± 38.2	325.0 ± 87.6^a^	16.4 ± 5.1	28.4 ± 3.6^a^	36.8 ± 14.6	75.6 ± 11.1^a^
*AUC*_(0-∞)_	ng/mL^a^h	511.9 ± 122.2	767.8 ± 150.8^a^	337.3 ± 150.7	569.8 ± 218.5^a^	17.6 ± 5.9	34.5 ± 9.8^a^	55.3 ± 22.9	75.9 ± 11.0^a^
*MRT*	h	5.1 ± 0.9	5.5 ± 1.3	10.5 ± 1.9	9.4 ± 2.2	3.9 ± 1.9	4.9 ± 1.8	2.3 ± 0.2	3.6 ± 0.7^a^
*t*_1/2z_	h	4.4 ± 1.6	5.6 ± 2.9	4.3 ± 3.4	9.8 ± 8.8	4.6 ± 2.9	8.1 ± 5.9	2.7 ± 0.8	3.0 ± 0.9
*T*_max_	h	0.9 ± 0.2	0.9 ± 0.1	1.2 ± 0.6	2.9 ± 2.1	0.3 ± 0.4	0.5 ± 0.4	1.8 ± 0.8	1.0 ± 0.3
*CL*z/F	L/h/kg	40.9 ± 9.5	27.1 ± 6.6^a^	68.1 ± 25.7	40.7 ± 18.9	1315.5 ± 693.1	616.3 ± 166.2	406.2 ± 209.5	267.9 ± 37.5
*V*z/F	L/kg	247.8 ± 68.2	200.7 ± 76.1	3307.5 ± 1876.8	438.4 ± 261.8^a^	6969.0 ± 3405.8	6174.7 ± 3089.6	1508.6 ± 839.6	1188.7 ± 466.4
*C*_max_	ng/mL	127.8 ± 56.0	218.6 ± 33.5^a^	16.8 ± 10.1	39.2 ± 5.9^a^	7.6 ± 0.7	16.8 ± 6.6^a^	13.1 ± 6.5	28.5 ± 12.3^a^

^a^ *p* < 0.05 compared with normal rats.

*AUC*, area under the plasma concentration–time curve; *MRT*, mean residence time; *t*_1/2z_, terminal half-life; *T*_max_, time at *C*_max_; *CL*z/F, clearance rate; *V*z/F, apparent volume of distribution; *C*_max_, peak concentration in plasma.

## 4 Discussion

The current cognition regarding the effects of diabetes mellitus on the pharmacokinetics and pharmacodynamics of antidiabetic drugs remains unclear. There are only substantially less data about the effects of diabetes mellitus on these properties of drugs for human, so the data obtained from the experimental animal models has extremely referential and practical values.

In this study, as shown in Table 4, the *AUC*_(0-t)_ of morin, morusin, umbelliferone and mulberroside A in diabetic rats were 717.3 ± 117.4, 325.0 ± 87.6, 28.4 ± 3.6 and 75.6 ± 11.1 ng/mL*h, compared with 501.3 ± 115.5, 116.4 ± 38.2, 16.4 ± 5.1 and 36.8 ± 14.6 ng/mL*h in normal rats (*p* < 0.05). Meanwhile, the *AUC*_(0-∞)_ of the target analytes obtained from diabetic rats were also significant increased (*p* < 0.05), compared with those obtained from the normal rats. Significant differences in maximum concentration (*C*_max_; 127.8 ± 56.0 vs 218.6 ± 33.5 ng/ml for morin, 16.8 ± 10.1 vs 39.2 ± 5.9 ng/mL for morusin, 7.6 ± 0.7 vs 16.8 ± 6.6 ng/mL for umbelliferone and 13.1 ± 6.5 vs 28.5 ± 12.3 ng/ml for mulberroside A) were observed between normal and diabetic rats (*p* < 0.05). Compared with normal rats, the *MRT* increased by 56.5% (*p* < 0.05) in the diabetic rats for mulberroside A. In addition, the *CL*z/F were decreased by 33.7%, 40.2%, 53.2% and 34.0% for morin, morusin, umbelliferone and mulberroside A in the diabetic rats, respectively. In addition, the bimodal phenomenon appeared in the mean plasma concentration-time curves of morusin in diabetic rats. Many factors would lead to the results such as the changes of gastrointestinal tract, liver and kidney function, local blood flow rate caused by diabetes. The results indicated that the pharmacokinetics of four target analytes in diabetic rats was significantly changed and the bioavailability was enhanced.

Diabetes mellitus is one of the most grievous problems threatening public health. Not only are antidiabetic drugs more widely used, but the pharmacokinetics of these antidiabetic drugs may also be changed due to the disease itself (Gwiltet al., 1991). Diabetes affects the metabolism of the three major nutrients, including protein, lipid and carbohydrate, and the systems that regulate biotransformation pathways of these nutrients also participates in the regulation of drug metabolism *in vivo* in many cases. The researches show diabetes could influence all processes of drugs in the body, such as the absorption, distribution, metabolism and excretion of drugs (Zini et al., 1990; Okabe and Hashizume, 1994; Cashion et al., 2004). For absorption, the significantly reduced in digestive tract blood flow caused by diabetes could be associated with the change of gastric pH, the prolongation of gastric emptying time and slowing of intestinal peristalsis. The differences in the absorption rate and bioavailability between normal and diabetic rats might depend on the above factors (Horowitz and Fraser, 1994; Horowitz et al., 1996). The higher level of circulating glucose in the blood would lead to non-enzymatic glycation of several proteins including albumin, which could affect the plasma protein binding rate of drugs (Day et al., 1979; Cohen et al., 2006). In addition, diabetes could affect drug metabolism due to the abnormal hepatic function caused by diabetes including non-alcohol steatohepatitis, macrovesicular steatosis, liver fibrosis/cirrhosis and focal fatty liver. It was also one of the important reasons for the changes in pharmacokinetics of the target analytes from Mori Cortex total flavonoid extract *in vivo* (Petrides et al., 1994; Wang et al., 2000). Diabetes nephropathy occurred frequently and it would influence the glomerular filtration, tubular secretion and tubular reabsorption, so the influence of excretion on the pharmacokinetics of drugs was also worthy of attention (Raine, 1995; Suzuki and Arakawa, 1995).

There were lots of factors that lead to pharmacokinetic changes in diabetes and further investigations would be required to reveal the underlying mechanisms for pharmacokinetics and pharmacodynamics of four target analytes in Mori Cortex total flavonoid extract in diabetic rats.

## 5 Conclusion

In summary, we have developed and validated a reliable, accurate and rapid UPLC-MS/MS method for simultaneous determination of morin, morusin, umbelliferone and mulberroside A in rat plasma. Moreover, this method was successfully applied for pharmacokinetic comparisons in normal and diabetic rats. The results would provide the pharmacokinetic rationale for the pharmacology and toxicology research of Mori Cortex.

## Data Availability

The raw data supporting the conclusion of this article will be made available by the authors, without undue reservation.

## Abbreviations

UPLC-MS/MS, ultra-performance liquid chromatography-tandem mass spectrometry; DM, diabetes mellitus; TCM, traditional Chinese Medicine; STZ, streptozotocin; FBG, fasting blood glucose; ESI, electrospray ionization source; MRM, multiple-reaction monitoring; QC, quality control; LLOQ, the lower limit of quantification; ULOQ, the upper limits of quantification; RSD, relative standard deviation; RE, relative error.
