# Supplementary figures and images for: Comparative pharmacokinetics of four major compounds after oral administration of Mori Cortex total flavonoid extract in normal and diabetic rats
Source: Front Pharmacol. 2023 Mar 1;14:1148332. doi: 10.3389/fphar.2023.1148332 (PMC10014546; doi:10.3389/fphar.2023.1148332)

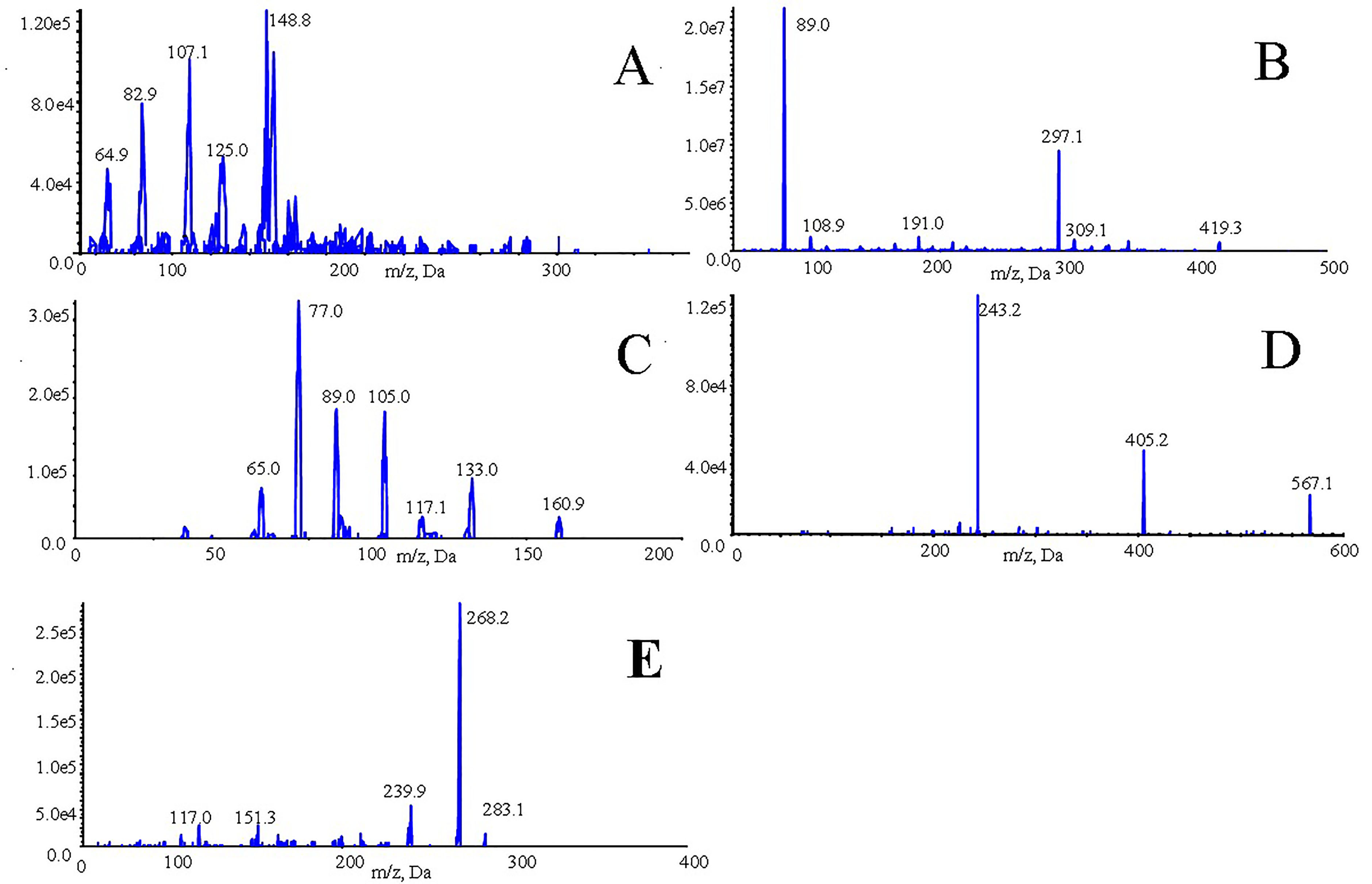

Supplement: Supplementary file 1 [file Image1.JPEG]
