# Supplementary material for: Comparative pharmacokinetics of four major compounds after oral administration of Mori Cortex total flavonoid extract in normal and diabetic rats
Source: Front Pharmacol. 2023 Mar 1;14:1148332. doi: 10.3389/fphar.2023.1148332 (PMC10014546; doi:10.3389/fphar.2023.1148332)
Supplement: Supplementary file 2 [file Table1.DOC]

**Supplementary Table S1**

The mean extraction recoveries and matrix effect of analytes in rat plasma (mean ± SD, *n* = 6).

| Analytes | Spiked concentration  (ng/mL) | Extraction recoveries(%) | Matrix effect(%) |
| --- | --- | --- | --- |
| morin | 3 | 88.5 ± 6.7 | 100.6 ± 10.2 |
|  | 40 | 98.3 ± 3.7 | 101.9 ± 5.9 |
|  | 800 | 101.4 ± 4.8 | 98.1 ± 2.7 |
| morusin | 3 | 100.4 ± 4.6 | 99.6 ± 9.4 |
|  | 40 | 102.2 ± 3.6 | 97.9 ± 6.7 |
|  | 800 | 104.3 ± 15.4 | 96.8 ± 10.4 |
| umbelliferone | 3 | 104.0 ± 11.3 | 96.0 ± 8.4 |
|  | 40 | 103.0 ± 4.0 | 93.8 ± 3.8 |
|  | 800 | 100.0 ± 4.9 | 95.5 ± 5.5 |
| mulberroside A | 3 | 109.0 ± 19.6 | 92.6 ± 10.3 |
|  | 40 | 96.7 ± 9.1 | 99.2 ± 6.5 |
|  | 800 | 100.0 ± 14.4 | 107.1 ± 12.0 |
| IS | 50 | 95.6 ± 3.8 | 101.2 ± 6.6 |

**Supplementary Table S2** Stabilities of analytes in rat plasma (*n* = 5).

| Analytes | Spiked concentration  (ng/mL) | Long-term stability | | Three freeze-thaw cycles | | Short-term stability | | Post-treatment stability | |
| --- | --- | --- | --- | --- | --- | --- | --- | --- | --- |
|  |  | RSD (%) | RE (%) | RSD (%) | RE (%) | RSD (%) | RE (%) | RSD (%) | RE (%) |
| morin | 3 | 9.8 | -6.4 | 9.8 | -4.6 | 5.9 | -0.9 | 4.7 | 2.1 |
|  | 40 | 3.0 | -9.4 | 1.5 | -12.8 | 6.1 | -8.9 | 4.9 | -9.6 |
|  | 800 | 1.3 | -4.8 | 6.4 | -8.4 | 4.2 | -3.7 | 6.4 | -7.2 |
| morusin | 3 | 7.0 | 4.7 | 8.5 | -2.5 | 10.9 | 2.8 | 9.0 | -1.9 |
|  | 40 | 10.4 | 1.0 | 6.1 | -9.2 | 10.2 | -1.5 | 6.5 | 0.9 |
|  | 800 | 4.4 | 4.1 | 7.2 | -5.6 | 5.1 | 1.2 | 4.7 | 0.6 |
| umbelliferone | 3 | 7.7 | -2.8 | 8.5 | -1.3 | 6.7 | -0.1 | 4.4 | -1.2 |
|  | 40 | 1.9 | 1.5 | 6.3 | -7.5 | 4.5 | -1.1 | 3.7 | 3.5 |
|  | 800 | 4.9 | -3.2 | 6.0 | -9.2 | 8.3 | -3.5 | 5.6 | -5.0 |
| mulberroside A | 3 | 7.6 | -4.1 | 9.0 | -0.5 | 8.1 | 2.5 | 15.1 | -1.5 |
|  | 40 | 9.2 | -3.0 | 7.5 | -2.3 | 8.6 | -5.3 | 8.0 | -0.5 |
|  | 800 | 6.4 | -8.6 | 5.1 | -9.0 | 10.3 | -4.7 | 11.0 | -5.2 |
